# Key Roles of Aliphatic Ligands over PbS Quantum Dots for Efficient Triplet Energy Transfer in a Hybrid TES-ADT/PbS System for Triplet–Triplet Annihilation Photon Upconversion

**DOI:** 10.1021/acs.jpcc.5c07155

**Published:** 2026-02-10

**Authors:** Naoyuki Nishimura, Zhilong Zhang, Victor Gray, James Xiao, Jesse R. Alladice, Akshay Rao

**Affiliations:** † Cavendish Laboratory, 2152 University of Cambridge, J. J. Thomson Avenue, Cambridge CB3 0HE, U.K.; ‡ Asahi-Kasei Corporation, 2767-11 Niihama, Shionasu, Kojima, Kurashiki 711-8510, Japan; § Department of Chemistry, Ångström Laboratory, 8097 Uppsala University, Box 532, Uppsala SE-751 20, Sweden

## Abstract

Photon upconversion (PUC) via triplet–triplet annihilation (TTA), using inorganic quantum dots (QDs) as triplet sensitizers, is a promising strategy for harvesting near-infrared photons due to the negligible energy loss associated with intersystem crossing in QDs. A TTA PUC system comprising 5,11-*bis*(triethylsilylethynyl)­anthradithiophene (TES-ADT) and lead sulfide (PbS) QDs effectively takes advantage of such advantages. Notably, in the liquid system, TES-ADT functions as both the triplet acceptor and TTA material, eliminating requirement of the conventional transmitter ligands that create energy losses via triplet energy transfer (TET). This unique dual-function presumably stems from a dynamic attach/detach mechanism: TES-ADT molecules detach to be free-floating molecules after accepting triplet energy and subsequently proceed with TTA. The emerging dynamic attach/detach mechanism is of general interest for hybrid systems of organic molecules and inorganic QDs; however, its mechanism remains elusive. Herein, modulation of aliphatic ligands over the QDs in TES-ADT/PbS QDs systems reveals that the affinity of TES-ADT molecules to the ligands can be a key factor for achieving efficient net TET via the dynamic attach/detach mechanism. In the steady-state PUC measurement, among the employed ligands (carbon numbers of the ligands 4C–18C), middle length ligands (8C and 12C) exhibited relatively high PUC and TET efficiency of up to 0.083% and 29%, respectively. Pump–probe transient absorption (TA) measurements suggest that the long ligand (18C) leads to the stacking of TES-ADT within the ligand shell, which reduces its net TET efficiency. Meanwhile, the 4C ligand presumably resulted in a lower affinity of TES-ADT to the shorter ligand, hampering the first step of TET. Conversely, ligands of length comparable to that of the ADT backbone (8C and 12C) most likely led to sufficient affinity to TES-ADT, allowing TES-ADT to detach/attach the PbS surface efficiently. Consequently, the insights obtained in this work will be clues for the development of inorganic–organic hybrid systems exploiting triplet energies.

## Graphical abstract

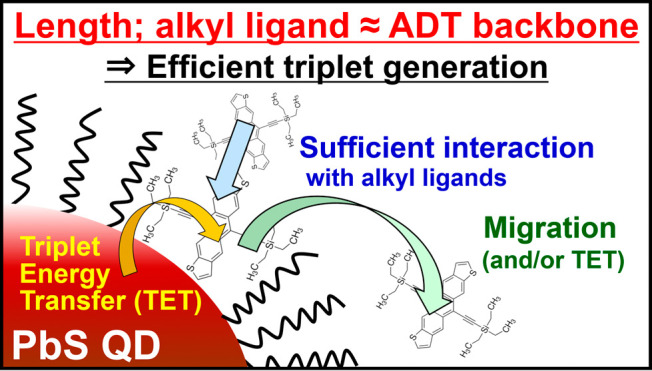

## 1 Introduction

Triplet energy transfer (TET) between inorganic quantum dots (QDs) and organic semiconductors has attracted considerable attention.^1,2^ One of the promising applications is triplet sensitization with the QDs for photon upconversion (PUC) via triplet–triplet annihilation (TTA).^3–27^ A key advantage of this approach is the ability of metal chalcogenide QDs, such as lead sulfide (PbS), to harvest near-infrared (NIR) photons, which are inefficiently captured by conventional triplet sensitizers based on heavy metal complexes. Notably, the absorption of NIR photons is crucial for boosting solar energy conversion efficiency of photovoltaics (PVs), in particular, crystalline silicon (c-Si) PVs (band gap ≈1.12 eV).^6,7^ Besides, using QDs as triplet sensitizers can be beneficial for reducing energy losses in the TTA PUC process as the energy required for an exchange between singlet and triplet excitons is negligible (i.e., within k*T* at room temperature), which would be an advantage for practical applications that require a large anti-Stokes shift.

However, QD triplet sensitizers in the TTA PUC system thus far have commonly created another energy loss in most of the systems with triplet transmitter ligands. As the TET is based on short-range Dexter energy transfer,^29–32^ which requires an orbital overlap between the donor and acceptor, the introduction of triplet transmitter ligands, which attach chemically onto the QDs surface with a functional group such as carboxylic acid (COO^–^) and can accept triplets, enables efficient extraction of energy from the QDs. Although this is a promising strategy to achieve highly efficient TET, this introduces an energy loss of several hundred meV, arising from the energy required for TET from the QDs to the transmitter ligand and the subsequent transfer to free-floating TTA materials.^22–25^ Furthermore, the relatively large triplet energies of the transmitter ligands (e.g., tetracene-based ones; *T*_1_ ≈ 1.2 eV) and TTA materials (e.g., rubrene; *T*_1_ = 1.14 eV) used in the conventional systems^13–17^ are not ideal to harvest NIR photons below the c-Si band gap. Therefore, development of both a novel strategy to reduce the energy loss in the TTA PUC process and a TTA material possessing a small triplet energy would be desired.

We have presented that a hybrid TTA PUC system consisting of 5,11-*bis*(triethylsilylethynyl)­anthradithiophene^7–10,33–38^ (TES-ADT; Figure 1a,b) molecules and PbS QDs (: TES-ADT/PbS QDs system) allowed TTA PUC with the smallest excitation energy (≈1.08 eV), which is below the c-Si band gap, at that time.^7^ In terms of utilizing the small excitation energy, this system takes advantage of both the nature of the PbS QD sensitizer harvesting NIR photons and the TES-ADT molecule that possesses a small triplet energy (*T*_1_: 1.08 eV) yet functions as a TTA material. In addition, this system successfully reduced the energy losses in the TTA PUC process compared to conventional systems, attributed to the minute driving energy of TES-ADT for proceeding with TTA (2T_1_–S_1_ ≈ 0). Importantly, the TES-ADT molecules perform a dual-function: both the triplet acceptor from the QD and the TTA material. Thus, no separate triplet transmitter ligand was needed in this system. This dual-function was attributed to the favorable interaction between TES-ADT and PbS, which allowed for close contact and a sufficient orbital overlap. The close contact was probably achieved through the chemical attachment of the thiophene moiety in TES-ADT to the PbS surface.

**Figure 1:**
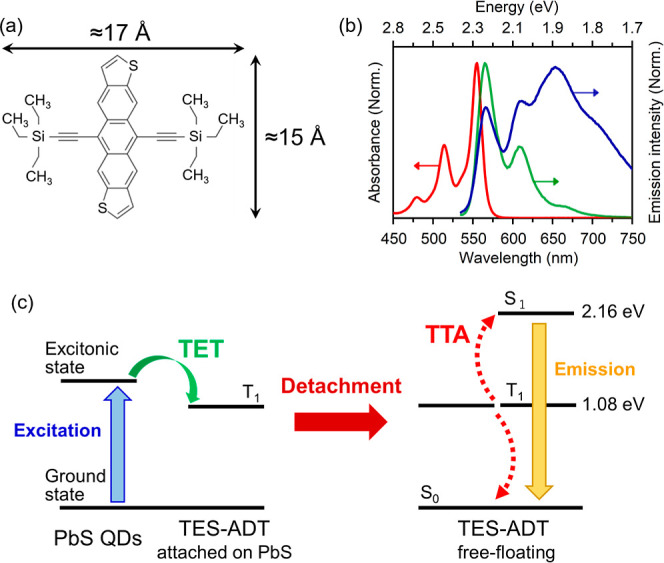
(a) Molecular structure of TES-ADT, (b) absorption (red) and emission spectra of TES-ADT solution (green: singlet with 0.2 mM, blue: excimer with 100 mM), and (c) TTA PUC scheme of the TES-ADT/PbS system via the dynamic attach/detach mechanism; adopted with permission from ref 7. Copyright 2019 Royal Society of Chemistry.

Furthermore, we have proposed that since thiophene possesses relatively weak electron negativity, the moderate interaction between TES-ADT and the PbS surface allowed for a dynamic attach/detach mechanism (Figure 1c): TES-ADT molecules detached to be free-floating molecules after accepting triplet energy from PbS QDs and subsequently proceeded with TTA (i.e., TES-ADT did not permanently attach on the PbS surface, unlike the conventional transmitter ligands).^7^ This dynamic attach/detach mechanism would be a key to the dual-function (i.e., both the triplet acceptor and the TTA material) and is of broad interest for hybrid systems based on organic molecules/inorganic QDs applicable to TTA PUC systems and/or singlet fission (SF) systems for photon doubling.^39^ However, the mechanism of TET in the dynamic attach–detach system, involving TES-ADT migration within the ligand shell, has remained elusive. Hence, further studies of the roles of aliphatic ligands over the QDs are desired.

This dynamic attachment/detachment mechanism may involve the interaction between the organic molecule and the native aliphatic ligands, which could then have a large impact on the TET and consequently the overall TTA PUC efficiency. For instance, we have revealed with small-angle scattering (SAXS) and small angle-neutron scattering (SANS) for PbS QDs with oleic acid ligands that some of the oleic acids do not chemically bind to the PbS surface but are present in the aliphatic ligand region via physisorption,^40^ which shows the importance of interaction with the alkyl ligands. This finding suggests that the interaction of the organic molecules with the aliphatic ligands may play a pivotal role within the net TET involving the dynamic attachment/detachment mechanism, whereas the effect of this physisorption-like interaction has not been previously discussed.

In this work, in order to obtain further clues about the net TET mechanism, we use steady-state PUC emission and pump–probe transient absorption (TA) measurements to study a set of TES-ADT/PbS QDs systems with varying aliphatic ligand length of the QDs. The present results strongly suggest that controlling affinity of the TES-ADT molecule to the aliphatic ligand has a large impact on the TET processes, most likely involving the dynamic attach/detach mechanism, and is vital for efficient TET in the TTA PUC process (Figure 2).

**Figure 2:**
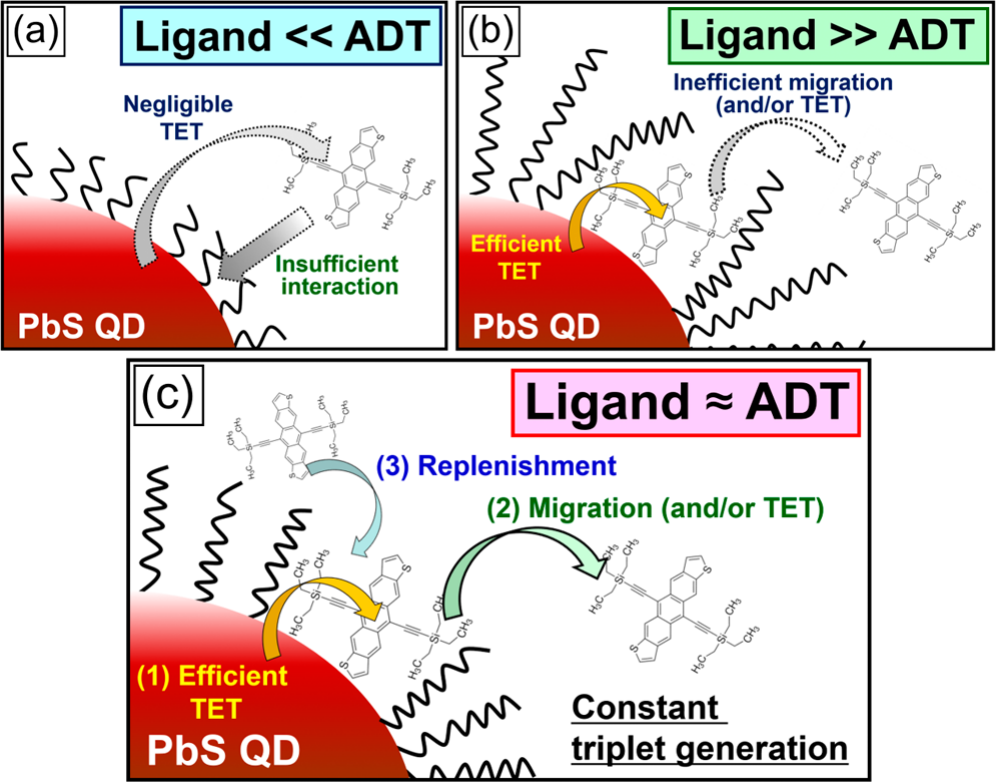
Schemes of TES-ADT/PbS systems with various alkyl ligand lengths in PbS QDs; (a) ligand ≪ ADT (e.g., C4 ligand), (b) ligand ≫ ADT (e.g., C18 ligand), and (c) ligand ≈ ADT (e.g., C8 and C12 ligand).

## 2 Experimental Methods

### 2.1 Materials

All original chemicals were purchased from Sigma-Aldrich and used as delivered.

### 2.2 Synthesis of PbS QDs

The synthesis of PbS QDs was carried out by a previously reported method.^7,41^ PbO (0.625 g, 2.8 mmol), oleic acid (3 mL, 9.5 mmol), and 1-octadecene (25 mL, 78 mmol) were placed in a 3-necked round bottomed flask and degassed under vacuum (<10^–2^ mbar) at 383 K for 2 h with stirring, forming a colorless solution. Subsequently, the flask was put under nitrogen flow and heated to the injection temperature at 398 K. In a nitrogen glovebox, a syringe was prepared containing 1-octadecene (13.9 mL, 43 mmol), diphenylphosphine (144 μL, 0.83 mmol), and hexamethyldisilathiane (296 μL, 1.4 mmol). The syringe containing the sulfur precursor was rapidly injected into the reaction flask and allowed to cool. The reaction mixture was transferred to an argon glovebox. The synthesized nanocrystals were twice purified through selective precipitation with ethanol/1-butanol and resuspension in hexane. The purified QDs were redispersed in toluene for storage at a concentration of 50 mg mL^–1^. The PbS QDs had an excitonic peak at 1.27 eV in the absorption.

### 2.3 Ligand Exchange of PbS QDs

For the synthesized PbS QDs, ligand exchanges to the desired carbon numbers of carboxylic acids were performed according to the literature with some minor modifications.^42^ In a nitrogen-filled glovebox, the QDs (50 mg mL^–1^ in toluene) were first loaded into separated vials and kept under magnetic stirring. The liquid-phase ligands (caprylic acid and butyric acid: 8C and 4C) were slowly added into the QD dispersion by using a micropipette. As the lauric acid (12C) ligand is in the solid state, it was first dissolved in toluene (∼50 mg mL^–1^). Typically, ∼0.1 mmol ligands were added to 100 mg of QDs. After the ligands were added, the QD dispersions were further stirred for about 30 min. The ligand-exchanged QDs were purified by adding extra toluene and acetonitrile, followed by centrifugation. The purification process was repeated by another two times to remove the residual ligands. The purified QDs were then dispersed in toluene at a concentration of 25 mg mL^–1^ and filtered before use.

For the prepared QDs, it was confirmed with TA measurements that the ligand exchanges did not affect the lifetime of each PbS QDs sample (Figure S1, Table S1; lifetimes of all PbS QDs: τ ≈ 2.3 μs). It should be noted here that a detectable lifetime change of PbS QDs after the ligand exchange to 8C ligands was observed in the previous paper where methanol was used for the purification of PbS QDs under the ligand exchange, while toluene–acetonitrile was employed for the washing solvent in this work. It is known that such methanol purification is much more prone to creation of defects over the PbS QDs than over toluene–acetonitrile.^43,44^ Therefore, the difference in the lifetime trend between this work and the previous work was most likely due to the difference of the solvents for the purification in the ligand exchange process, and the PbS QDs used in this work should possess significantly fewer defects, as exhibited in the TA lifetime (Figure S1, Table S1). Hence, the present series of PbS QDs are suitable for investigating and discussing the effects of the affinity between the TES-ADT molecule and the aliphatic ligands covering the QDs.

### 2.4 Steady-State Absorption and Emission Measurement

Absorption spectra were measured on degassed toluene solutions containing TES-ADT and/or PbS QDs in a 1 mm path length quartz cuvette using a spectrometer (UV3600-Plus, Shimadzu). Emission spectra was measured using a spectrograph (Shamrock SR-303i, ANDOR) with a CCD camera (Andor iDus DU420A Si CCD, ANDOR), calibrated for spectral sensitivity of the detector at each wavelength. The samples filled in a 1 mm path length quartz cuvette were excited by a 520 nm CW laser (Thorlabs) from the side facing the detector. The emitted light passed through a 500 nm long pass filter (Thorlabs) before the detector.

### 2.5 PUC PLQY Measurement

In all experiments, degassed 100 mM TES-ADT toluene solutions were used for the TES-ADT/PbS systems. PUC PLQY was measured with a detector constructed with a spectroscopy camera (Andor iDus DU420A Si detector, ANDOR) coupled to a spectrograph (Shamrock SR303i, ANDOR) and samples consisting of TES-ADT and PbS QDs filled in a 1 mm path length quartz cuvette excited by a CW laser. The QY (Φ_UC_) was calculated with the following eq 1

1 $$\phi_{UC}=\phi_{r}\frac{\left(1-10^{-A_{r}}\right)}{\left(1-10^{-A_{x}}\right)}\frac{F_{x}}{F_{r}}\frac{{\left(\eta_{x}\right)}^{2}}{{\left(\eta_{r}\right)}^{2}}$$

where Φ_*r*_ is the QY of emission from the reference, *A*_*i*_ is the absorption at the excitation wavelength, *F*_*i*_ is the integrated emission, η_*i*_ is the refractive index of the solvent, and subscripts *x* and *r* designate the sample and reference, respectively. Rhodamine B in degassed ethanol with an excitation at 520 nm was applied as the reference (Φ_*r*_ = 0.97) in this work. The emitted light (*F*_*x*_) was collected between 545 and 800 nm with an excitation at 1064 nm from a CW laser (Thorlabs) passing through a 1000 nm long pass filter (for PbS QDs) from the side facing the detector. The emission from the sample passed through an 850 nm short pass filter before the detector. The excitation power was controlled to 240 W cm^–2^ with an ND filter.

### 2.6 TA Measurement

TA spectra were recorded over time delays from 1 ns to 300 μs with a pump of 200 nJ/pulse at 1064 nm (for PbS QDs in the presence or in the absence of TES-ADT) and a probe pulse covering between 850 and 1000 nm. The pump triggered with the probe pulse and delayed with electrical control was generated from a Nd:YVO_4_ laser (Picolo laser; InnoLas Laser). The probe pulse was generated from a home-built noncolinear optical parametric amplifier with a BBO single crystal and a pulsed laser from a Ti: sapphire amplifier system (Soltice; Spectra Physics) operating at 1 kHz. The probe beam was split into two before the sample filled in a quartz cuvette with a path length of 1 mm, and then, the probe beam and the pump beam were overlapped on the sample adjacent to another probe beam passing through the sample as the reference. The beams after the sample were focused into as imaging spectrometer (Shamrock SR 303i; Andor) and detected by a pair of linear image sensors (G11608, Hamamatsu Photonics) driven and read out at full laser repetition rate by a custom-built board from Stresing Entwicklungsbüro. In all measurements, every second pump shot was omitted electrically in order to obtain the fractional differential transmission (Δ*T*/*T*). The Δ*T*/*T* of the probe was calculated for each data point once 1000 shots had been collected.

### 2.7 Estimation of Ligand Length

The length of the alkyl ligand of PbS QDs (ligand) was estimated with previously reported values^32–34^ along with the following equation (eq 2)­

2 Ligand(Å)=LC−O+1.25NC

where LC–O and NC represent the bond length of C–O (2.3 Å) and the numbers of carbon in each ligand, respectively. The value of 1.25 Å was used for contribution of each C–C bond to the carbon chain length in this equation.^45–47^

It should be noted here that the previous report indicates that in the solid state, formation of the “wet hair structure” was expected to occur with long alkyl chain ligands and will decrease the ligand layer thickness from the alkyl chain length.^42^ However, the condition employed in this work was in a toluene solution that possesses a good affinity to the alkyl ligands, and so, formation of the wet hair structure decreasing in the ligand layer thickness is unlikely to happen. In addition, the existence of ligand molecules stacked in the ligand layer with unbound functional groups,^40^ which we recently found, will prevent curving the alkyl chain and will prevent bending of the alkyl chains. Hence, for considering the interaction between the alkyl ligands and TES-ADT molecule in this work, using the value of alkyl ligand length is appropriate.

## 3 Results and Discussion

### 3.1 Steady-State PUC and TET Efficiencies of TES-ADT/PbS QDs Systems

All prepared TES-ADT/PbS QDs systems generated PUC emission at around 660 nm with an excitation at 1064 nm, which is in line with the previous report^7–10^ (Figure S2) The observed red shift in the PUC emission, relative to the singlet TES-ADT emission, is attributed to excimer formation.^7^ (Figure 1b). Figure 3 depicts dependence of PUC photoluminescence quantum yield (PLQY) and TET efficiency with a change in the ligand length (i.e., the number of carbons in each ligand). The TET efficiencies were estimated with the following equation (eq 3)­

3 $$\Phi_{UC}=1/2\Phi_{ISC}\times \Phi_{TET}\times \Phi_{TTA}\times \Phi_{FL}$$

**Figure 3:**
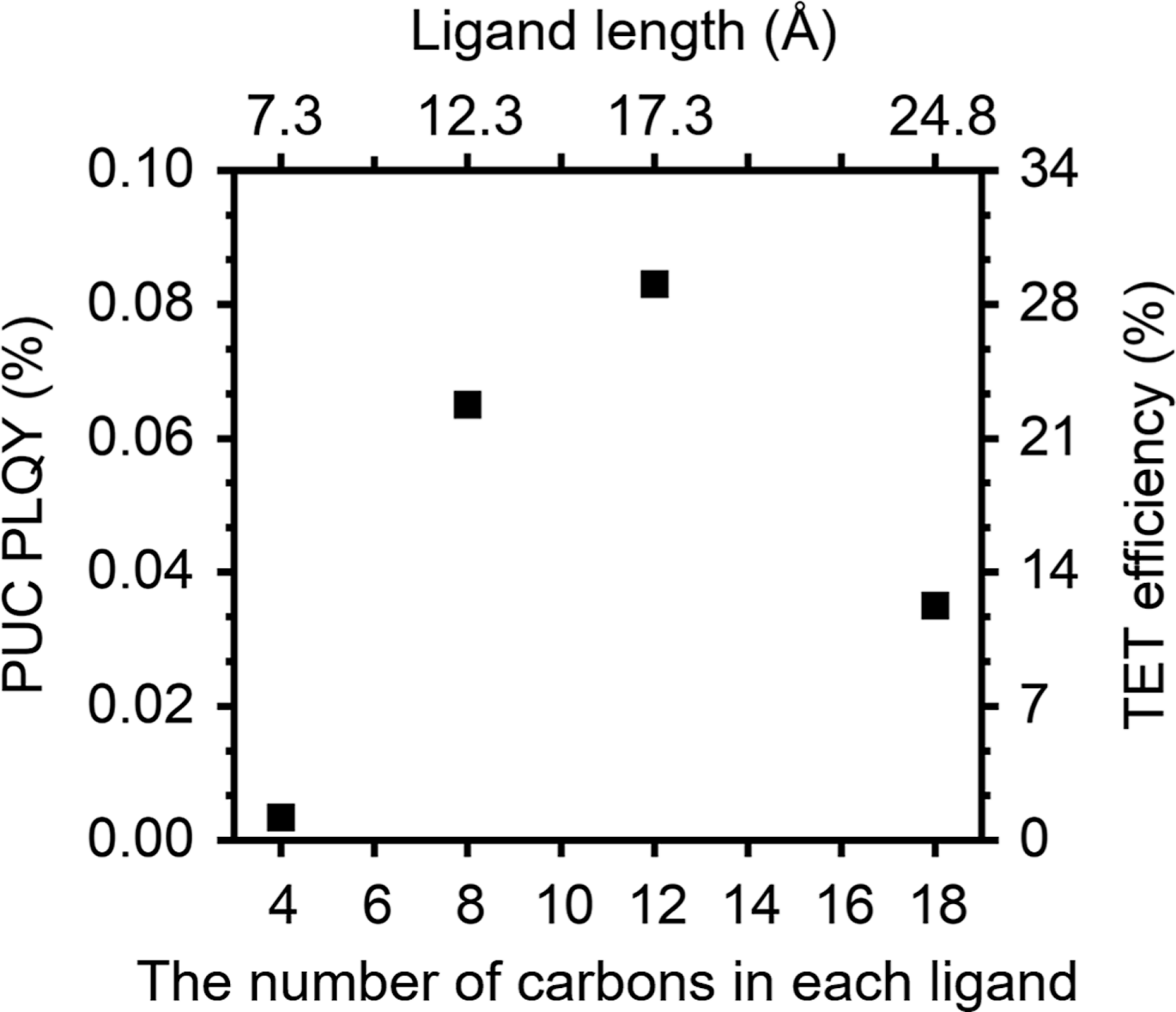
PUC PLQY and TET efficiencies of the TES-ADT (100 mM)/PbS QDs (2.5 mg) system excited at 1064 nm along with changing carbon chain length in the ligands.

The Φ_UC_, Φ_ISC_, Φ_TET_, Φ_TTA_, and Φ_FL_ are the PUC PLQY, intersystem crossing (ISC) efficiency, TET efficiency, TTA efficiency, and PLQY of TES-ADT emission with the direct singlet excitation, respectively. The maximum value of Φ_UC_ in this work is 50% due to the two-photon process. In the previous work, the values of Φ_ISC_ = 100%, Φ_FL_ = 2.9% (in 100 mM TES-ADT toluene solution), and Φ_TTA_ = 20% were obtained,^7^ and thus, we can estimate the Φ_TET_ value from the obtained Φ_UC_. We note that the Φ_TET_ estimated here means the efficiency of the net TET, consisting of both TET from PbS QDs to TES-ADT and the subsequent process for the triplet to be free-floating.

On decreasing the ligand carbon number from 18 to 12, corresponding to a change in the ligand length from 24.8 to 17.3 Å, the Φ_UC_ and Φ_TET_ increased more than 2-fold from Φ_UC_ = 0.035% and Φ_TET_ = 12% to Φ_UC_ = 0.083% and Φ_TET_ = 29% (Figure 3). A further decrease in the carbon number from 12 to 8 corresponding to a change in the ligand length from 17.3 Å to 12.3 Å led to a slight decrease in the Φ_UC_ and Φ_TET_ to 0.065% and 22%, respectively, which is the same trend as in the previous report.^8^ Meanwhile, the 4C sample (with a ligand length of 7.3 Å), which is the shortest ligand in this work, resulted in negligible efficiencies (Φ_UC_ = 0.0034% and Φ_TET_ = 1.2%), which was not investigated in the previous work.^8^

The high TET efficiencies of 12–28% obtained with 8C–18C PbS QDs sensitizers are 3 orders of magnitude higher than previous reports of alkyl-chain-coated QDs without surface-anchored transmitter ligands (Φ_TET_ up to 0.02%).^48,49^ Hence, this trend supports the idea that close contact and orbital overlap between TES-ADT molecules and PbS QDs were successfully formed. Moreover, if TES-ADT did not form close attachment onto the PbS surface and the TET occurred via transfer through the aliphatic ligands instead, the efficiency would be dependent on the ligand length, and thereby, the shorter ligand length would result in higher efficiencies. However, as is clear from the data in Figure 3, this is not the case, a finding consistent with the formation of close contact between TES-ADT and the PbS QDs.

On the other hand, the minimal efficiencies of the sample with the 4C ligand strongly suggest that with the 4C sample, TES-ADT molecules could not approach the QD surface effectively. Taking into account the ligand lengths, this trend would provide clues about the close attachment of TES-ADT onto QDs. While the length along the ADT backbone was approximately 15 Å ^35,36^ (Figure 1a), the length of the 4C ligand was estimated to be 7.3 Å, which is almost half of the TES-ADT backbone, and hence, interaction of the TES group with the 4C ligand would be considerably less compared to that with a longer ligand such as 8C (12.3 Å). Thus, it is strongly suggested that the lack of affinity of the 4C ligands to TES-ADT molecules did not allow the close attachment of TES-ADT to the PbS surface and thereby resulted in the drop of the TET and the corresponding PUC efficiency (Figure 2a). It should be emphasized that given the low electronegativity of the thiophene moiety in the TES-ADT molecule, the substantial contribution of the affinity between TES-ADT and the aliphatic ligands to the successful close attachment of TES-ADT onto the PbS surface is plausible. Consequently, sufficient interaction between aliphatic ligands of QDs and the triplet acceptor that does not have a strong functional group can be vital in order for the acceptor molecules to approach the QDs closely and thereby render efficient TET feasible.

Moreover, proper affinity control between ligands of QDs and the triplet acceptor can be an effective strategy to enhance TET and TTA PUC efficiency, which is represented by comparing the 12C and 8C samples to the original 18C sample; the further clues will be provided by the TA measurement.

It is highly noteworthy here that two theories relevant to the effects of the aliphatic ligands over the QDs on Φ_TET_ have been proposed. The first factor is the dielectric constant for a solid-state system; in the previous report about a solid-state TTA PUC system consisting of an aggregated QDs layer that functioned as the triplet sensitizer (i.e., a solid-state rubrene/monolayer PbS QDs PUC system), a decrease in the aliphatic ligand length of QDs led to an increase in the dielectric constant of the QDs layer, which was estimated with the Bruggeman model^50^ that provides dielectric constant for the average value of the aggregated QD layer consisting of the QDs core (e.g., PbS regime) and the alkyl ligand, and thereby, the resulting large dielectric constant reduced its Φ_TET_ and Φ_UC_. However, this estimation was for such solid-state systems where the QDs densely aggregated, but it is not suitable for liquid systems involving the PUC system in the present work. This is because the estimation with the Bruggeman model^50^ is not proper for the solution system where the QDs dispersed with much lower density compared to the solid system. Although the change in the dielectric constant might partly contribute to the observed significant decrease in Φ_TET_ and Φ_UC_ of the 4C sample (Figure 3), this effect for the QDs-dispersed solution system should be considerably less than that in the case of the solid-state system. Consequently, the lack of affinity of TES-ADT molecules to the short 4C ligands most likely was the main cause for the decreases in the efficiencies.

The second theory is about defect formation; it has been inferred that defects formation via the ligand exchange to shorter ligands (only 8C and 12C were employed in the previous work), which can be represented by a change in the QDs lifetime, would also contribute to the decrease in the TET efficiency because the triplets trapped in the defect level could be too deep to proceed with the TET.^8^ This factor might have contributed to part of the Φ_TET_ drop, whereas the ligand exchange to 4C in the present work resulted in almost the same lifetime as that of the native ligand (18C), as shown in Figure S1, despite the 10 times lower Φ_TET_ of the 4C sample than that of the 18C sample (Figure 3). We note that the unchanged lifetime even for the short 4C ligand QDs is consistent with the choice of solvent for the ligand exchange process^43,44^ (see the Experimental Methods section). Hence, this 4C result strongly suggests that factors other than defect formation mainly caused this steep drop of the Φ_UC_ and Φ_TET_ for the 4C sample, and the lack of the affinity is the most plausible in light of discussions above.

### 3.2 Investigation of the TET Dynamics by Pump–Probe TA Measurement

To investigate the TET dynamics in more detail, pump–probe TA measurements were carried out. Figure 4a (4b: zoomed-in) displays TA spectra of the PbS QDs with the 12C ligands in the presence of 100 mM TES-ADT with an excitation at 1064 nm. A positive peak observed at 980 nm is assigned to the ground state bleach (GSB) of the QDs^50–54^ at early delay times. The intensity of this GSB signal subsequently decreases. After that, photo-induced absorption (PIA) at around 920 nm that is assigned as the triplet feature (i.e., T_1_ → T_*n*_ transition) of TES-ADT^7^ appeared at 10–20 μs (Figure 4b) and finally disappeared at 100–200 μs. We here note that in the previous study, the triplet feature of TES-ADT at the 920 nm PIA signal was confirmed via direct S_1_ excitation of a dense TES-ADT solution that proceeded with singlet fission forming its triplets (i.e., S_1_ → 2T_1_).^7^ Thus, the existence of a clear peak at around 920 nm is evidence of TET from the QDs to TES-ADT. It is also noted that we choose a nano-second-ordered pulse laser excitation for this experiment to circumvent occurrence of the two-photon absorption because pulse excitations with short pulse duration (e.g., subpicosecond pulses) likely cause two-photon absorption.

**Figure 4:**
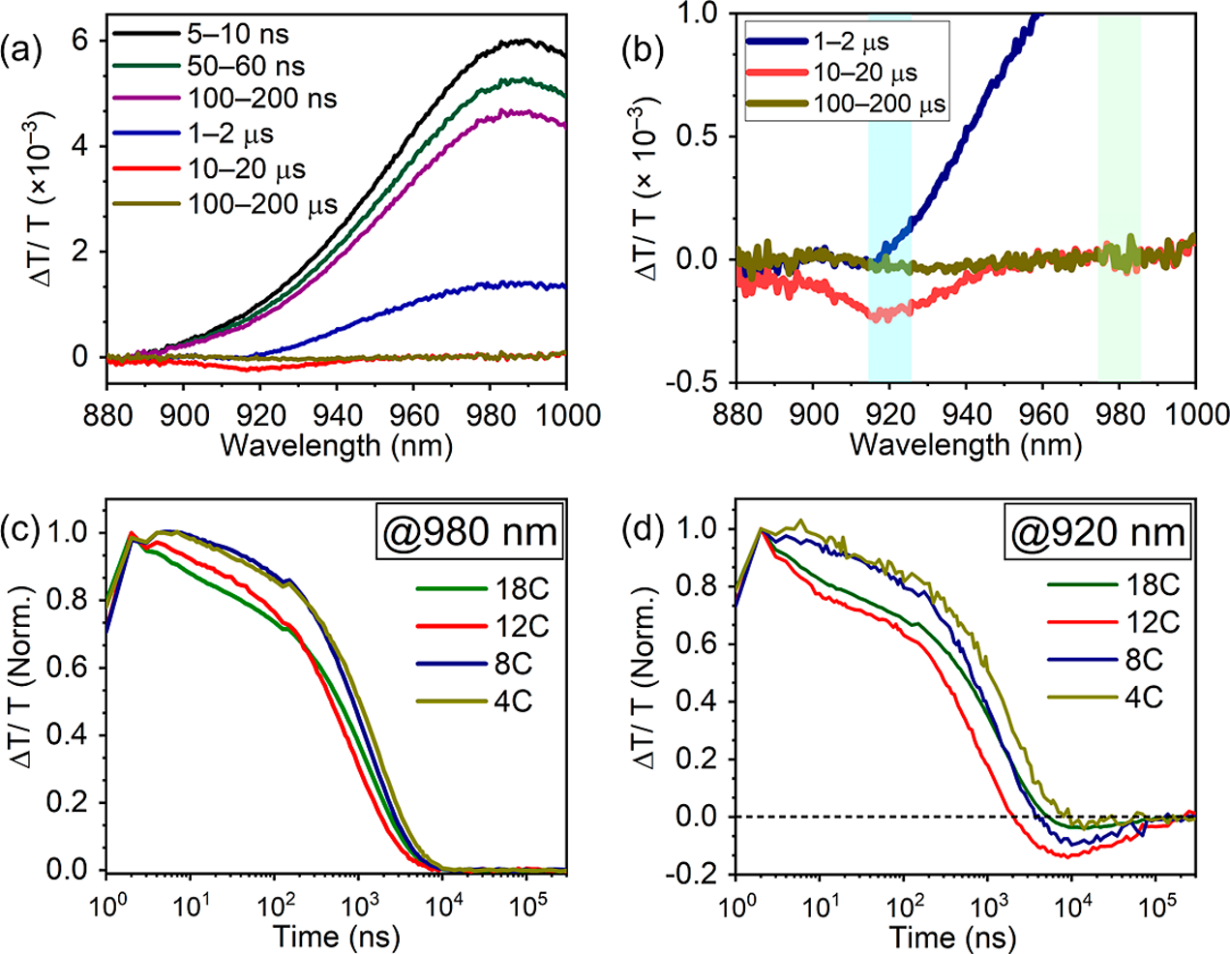
(a) ((b) zoomed-in) TA spectra of the QDs with 12C ligands in the presence of 100 mM TES-ADT with an excitation at 1064 nm. Kinetics of TA spectra of TES-ADT/PbS QDs systems with the various ligand lengths from 4C to 18C (c) at 980 nm and (d) at 920 nm.

To follow the kinetics of PbS QDs decay and TES-ADT triplet growth, TA kinetics at 980 and 920 nm are depicted in Figure 4c,d, respectively. The TA kinetics changed with changing ligand length, and decay trend changes were observed. It is noteworthy that such decay trend changes were not observed with a rubrene-based system using the same PbS QDs (Figure S3 and Table S2). Rather, the rubrene-PbS QDs system showed negligible quenching of the PbS QDs in the presence of rubrene, which is consistent with the inefficient TET previously reported (up to 0.02%).^48,49^ Thus, it was confirmed that multiple decays were derived from the intrinsic interaction between TES-ADT and PbS QDs.

In the TA signal decay at 920 nm (Figure 4d), involving both quenching of PbS QDs and triplet generation of TES-ADT, negative Δ*T*/*T* values were clearly observed after 1 μs, indicating triplet generation trends of TES-ADT. The order of magnitude of the triplet generation was 12C > 8C > 18C > 4C, which is in line with the order of the PUC efficiencies, corresponding to their steady-state TET efficiencies (Figure 3). Therefore, the overall TET transfer proceeded along with this order, whereas the TA decay trends differ among samples with different aliphatic ligand lengths, providing clues for functional mechanisms of the TES-ADT/PbS QDs system.

While 8C and 4C samples showed almost constant TA signal decays, 18C and 12C samples exhibited a nanosecond order decay in the initial stage of the TA decay at 920 nm (Figure 3d). In addition, although the magnitude was different from the case of the TA decay at 980 nm, the TA signal decays at 980 nm showed similar trends (Figure 4c). Thus, a boundary between 12C (17.3 Å) and 8C (12.3 Å) for the functionality of the TES-ADT/PbS QDs system was anticipated and can be related to the size of the TES-ADT backbone (15 Å).

The previous study^7^ revealed that TES-ADT molecules chemically attach on PbS QDs with the 18C ligands and extract triplets from the PbS QDs within a nanosecond order range, which is consistent with the time order for TET via the orbital overlap, as previously reported.^5,7,14–17,52–57^ Hence, the observed initial TA decays for 18C and 12C samples could be attributed to the triplet extraction of TES-ADT molecules from PbS QDs via the chemical attachment. This character may represent that the sufficiently long aliphatic ligands, relative to the TES-ADT backbone (15 Å), provide sufficient affinities of TES-ADT molecules to PbS surfaces and thereby allow the chemical attachments of TES-ADT to PbS QDs. The noncontinuous fast decay was attributable to the limited coverage of TES-ADT molecules on the PbS surface as each TES-ADT molecule is capable of accepting only one triplet exciton. However, the 8C sample also exhibited an efficient overall TET character, even more efficient than the 18C sample, and thus, the 8C sample should also involve the orbital overlap TET from PbS QDs to TES-ADT. Meanwhile, the overall TET process in TTA PUC is stepwise as follows (i) TET from triplet sensitizers (i.e., PbS QDs in this work) to transmitter materials (i.e., TES-ADT in this work) and (ii) detachment of the transmitter materials from the PdS surface to be free-floating TTA materials via the dynamic attachment/detachment mechanism. Therefore, the difference in the latter step (process (ii)) in the TES-ADT/PbS QDs system presumably led to the different TA decay trends between 18 and 12C and 8C samples.

The previous work also proposed that the rate-determining step in TES-ADT/PbS QDs with the 18C ligand was estimated to be of microsecond order and probably associated with (ii) gradual detachment of TES-ADT from the PbS surface involving migration of the TES-ADT molecules (Figure 2b).^7^ This is because each TES-ADT molecule is capable of accepting only one triplet exciton, and subsequently, in order to accept other triplets, the TES-ADT has to utilize the triplet exciton via TTA. The gradual detachment of TES-ADT from the PbS surface will be significant if the aliphatic ligands are longer than the TES-ADT backbone. Hence, for samples with 18C (24.8 Å) and 12C (17.3 Å), longer than the TES-ADT backbone (15 Å), the TES-ADT diffusion from the ligand regime following the detachment of TES-ADT from the PbS surface can be the rate-determining step in the overall TET process. In other words, the aliphatic ligands longer than the TES-ADT backbone (15 Å) can cause stacking of TES-ADT in the ligand regime (Figure 2b), while the shorter aliphatic ligands can address this issue. Moreover, the time range difference between (i) nanosecond-order TET from PbS to TES-ADT and (ii) TES-ADT diffusion following the TES-ADT detachment presumably created the clear initial decay in the TA decay (Figure 4d). However, the 8C ligand (12.3 Å) is shorter than the TES-ADT backbone (15 Å) and thereby can eliminate the TES-ADT diffusion process (step (ii)), resulting in its seamless TA decay (Figure 4d). Moreover, in the 12C sample, the faster decay after the initial nanosecond order decay, compared to the 18C sample (Figure 4d), suggests that the shorter aliphatic chain allowed faster moving of TES-ADT molecules away from the ligand regime and thereby facilitated net TET processes, leading to the higher net TET efficiency (Figure 3; 12C: 29%, 18C: 12%).

It is noteworthy here that process (ii) might be the TET process from the TES-ADT attached on the PbS surface to free-floating TES-ADT molecules (permanent attachment mechanism), yet the proposed dynamic attachment/detachment mechanism is far more plausible. This is because although a number of studies on TET using the 18C-modified metal chalcogenide QDs with the transmitter ligands smaller than TES-ADT (e.g., tetracene- or anthracene-based one), which are more likely covered by the 18C ligands, have been investigated thus far, the decays with such a distinct boundary in the initial TA decay has not yet been reported, expect for this system.^28,54–63^ Moreover, although the 12C sample in this work also exhibited the distinct initial TA decay, similar to the 18C sample, the size difference between the 12C chain (17.3 Å) and TES-ADT backbone (15 Å) is minute, unlike for the 18C sample (24.8 Å), and thus, even if TES-ADT molecules permanently attach onto the PbS surface (permanent attachment mechanism), flee-floating TES-ADT molecules can frequently approach the transmitter TES-ADT molecule on PbS and thereby will not create such a distinct initial TA decay. Therefore, results obtained in this work support the occurrence of the dynamic attachment/detachment mechanism.

The elimination of the TES-ADT stacking at the PbS surface in step (ii), in principle, is advantageous for efficient overall TET via avoidance of the quenching triplet in TES-ADT. This is because the orbital overlap of the triplet excited TES-ADT with PbS QDs leads to a short triplet lifetime of TES-ADT via the heavy atom effect of the Pb cations.^16,17,28,29^ Indeed, the average lifetimes of the TA decay at 980 nm, which may represent lifetimes of PbS QDs (Figure S4 and Table S3; see the Supporting Information for the details), suggest quenching of triplets in TES-ADT using long aliphatic chains. Although the 18C sample exhibited lower steady-state TET efficiency (12%) than the 8C sample (22%), a shorter lifetime of PbS QDs (18C: 1195 ns, 8C: 1440 ns) was observed. This is presumably because the stacking of TES-ADT molecules on the PbS surface shortened the triplet lifetime via the heavy atom effects, and thereby, the triplets were quenched even though they were not used for TTA PUC. Subsequently, the stacked TES-ADT molecules could accept another triplet from PbS, and ultimately, PbS quenches were likely facilitated. This character could contribute to the decrease in the net TET efficiency for the 18C sample, compared to the 8C sample.

Meanwhile, the 12C sample exhibited a slightly higher steady-state TET efficiency (29%) than the 8C sample (22%), even though it showed the rate-determining diffusion process of TES-ADT molecules (Figure 4d, scheme: Figure 2b). This character highlights the crucial role of aliphatic chains, providing sufficient affinity to TES-ADT molecules; the longer aliphatic chain is more advantageous for close attachment of TES-ADT molecules on the PbS surface, and 12C ligands allowed more frequent and/or close interaction of TES-ADT molecules to PbS surfaces compared to 8C, resulting in a higher net TET efficiency. The importance of the aliphatic chain function that provides sufficient affinity to TES-ADT molecules is also in accordance with the low net TET efficiency of the short-chain 4C sample (1.2%) and the intrinsically low electronegativity of the thiophene moiety in the TES-ADT molecule, which likely requires support in attaining its close attachment to PbS surfaces.

Consequently, the TA measurements suggested the instrumental roles of the aliphatic chain length of the QD ligands (Figure 2) and supported the occurrence of the dynamic attachment/detachment mechanisms. The TA decay trends suggested that the long aliphatic ligand (e.g., 18C) led to the hindrance of triplet migration away from the PbS QDs through TES-ADT detachment, reducing its net TET efficiency (Figure 2a). Besides, the inefficient migration may also have led to a reduced triplet lifetime, caused by the heavy atom effect, which is also detrimental for the net TET and TTA PUC process. Meanwhile, the appropriate aliphatic ligand length (8C: 12.3 Å, 12C: 17.3 Å), which is similar to the length of the ADT backbone (15 Å), allowed both the fast extraction of triplet from PbS QDs to TES-ADT and the efficient triplet migration away from the QD, leading to relatively high net TET efficiency (Figure 2c).

## 4 Conclusion

In conclusion, we revealed with steady-state PUC emission measurement and pump–probe TA measurement that in the TES-ADT/PbS QDs system, the affinity of the TES-ADT molecule for the aliphatic ligands on the PbS QD surface plays a pivotal role in the TET from PbS QDs to TES-ADT molecules and the subsequent triplet migration away from the PbS (Figure 2). Furthermore, the insights obtained in this work contribute to a comprehensive understanding of the TES-ADT/PbS system; the close attachment between TES-ADT and PbS QDs, which could not be feasible with conventional TTA materials such as rubrene, was enabled by the weak electric negativity of the thiophene moiety in TES-ADT, most likely with substantial assistance from the physisorption of TES-ADT to the aliphatic ligand over the QDs (Figure 2).

With a decrease in the number of carbons in the aliphatic ligand on PbS QDs from 18 to 12 or 8, TET efficiency and PUC PLQY increased up to two times (TET: 29%, PLQY: 0.083% with the 12C ligand). However, with the shortest 4C ligand, the efficiencies dropped to negligible values, most likely due to the too short length of the 4C ligand (7.3 Å) to provide the sufficient affinity for the TES-ADT molecule (ADT backbone: 15 Å) to reach the PbS surface (i.e., 4C was not enough to gain sufficient affinity of TES-ADT with the aliphatic ligands via physisorption) (Figure 2a). Thus, a ligand length similar to the ADT backbone, which provides proper affinity of TES-ADT to the aliphatic ligands, is the key to the efficient net TET in the TTA PUC process.

The pump–probe TA measurements provide further insights into the TET mechanisms (Figure 2) and support the occurrence of dynamic attachment/detachment mechanisms. The TA decay trends differ between the aliphatic ligands of length longer and shorter than the TES-ADT backbone (15 Å), supporting the contribution of aliphatic ligand lengths to the TET mechanisms. While longer aliphatic ligands are advantageous in attaining close attachment of TES-ADT molecules onto PbS surfaces, gradual diffusion of TES-ADT molecules away from PbS surfaces presumably became the rate-determining step in their net TET process. The stacking of TES-ADT molecules in the ligand regime also caused a decrease in triplet lifetime of TES-ADT due to the heavy atom effects of Pb in PbS. Thus, an excessively long aliphatic ligand can decrease the net TET efficiency, which was observed for the 18C sample. Meanwhile, the 12C sample exhibited higher net TET efficiency than the 8C sample, suggesting that the most crucial role of the aliphatic ligands is providing sufficient affinity to TES-ADT molecules, allowing their close attachment to the PbS surfaces and highlighting its importance.

Consequently, this work strongly suggests that in solution systems, controlling the affinity of organic semiconductor molecules to the aliphatic ligands covering inorganic QDs can play an important role, yet physisorption of organic molecules to the aliphatic ligands has seldom been discussed thus far. Therefore, the insights presented here provide guidance for the future design of hybrid systems for applications in exciton fission and fusion (i.e., singlet fission and TTA PUC), and they should stimulate further research in this area.

## Supplementary Material



## Notes

Kinetics data fitting, lifetimes of pristine PbS QDs after the ligand exchanges, TTA PUC emission from the TES-ADT/PbS QDs system, lifetimes of PbS QDs in the presence of tubrene molecules, and quenching of PbS QDs in the presence of TES-ADT (PDF)
