# Supplementary material for: Key Roles of Aliphatic Ligands over PbS Quantum Dots for Efficient Triplet Energy Transfer in a Hybrid TES-ADT/PbS System for Triplet–Triplet Annihilation Photon Upconversion
Source: J Phys Chem C Nanomater Interfaces. 2026 Feb 10;130(7):2616–24. doi: 10.1021/acs.jpcc.5c07155 (PMC12927020; doi:10.1021/acs.jpcc.5c07155)
Supplement: Supplementary file 1 [file jp5c07155_si_001.pdf]

Supporting Information

Key Roles of Aliphatic Ligands over PbS Quantum Dots for Efficient Triplet Energy Transfer in a Hybrid TES-ADT/PbS System for Triplet-Triplet Annihilation Photon Upconversion

Naoyuki Nishimura [†, ‡], *Zhilong Zhang* [†], *Victor Gray* [†, §], *James Xiao* [†], *Jesse R. Alladice* [†], *Akshay Rao* [†, *]

† Cavendish Laboratory, University of Cambridge, J. J. Thomson Avenue, Cambridge, CB3 0HE, United Kingdom

‡ Asahi-Kasei Corporation, 2767-11 Niihama, Shionasu, Kojima, Kurashiki, Okayama, 711-8510, Japan

§ Department of Chemistry, Ångström Laboratory, Uppsala University, Box 532, SE-751 20 Uppsala, Sweden

Kinetics data fitting

Data fitting for TA lifetimes (τ) for Figure S1, S2, and S4 were carried out with Origin® software. The TA decays in Figure S1 and S2 were fitted with a mono exponential decay. The TA kinetics for Figure S4 were fitted with triple exponential decays ($\tau_{1,2,3}$), and its average value ($\tau_{av.}$) was calculated as the amplitude-weighted mean (i.e., $(A_1\tau_1+A_2\tau_2+A_3\tau_3) / (A_1 + A_2 + A_3)$).

For TES-ADT/PbS QDs systems (Figure 4), exponential fitting could be difficult to differentiate their kinetics parameters. This is because in this system, the functionality of TES-ADT is the attachment/detachment mechanism that is stepwise process comprising steps with significantly different time scales, whereas exponential fitting cannot describe such non-continuous system. Thus, we calculate its average lifetime ($\tau_{av.}$), which may suggest their decay trends, and discussed it as a support.

Lifetimes of pristine PbS QDs after the ligand exchanges

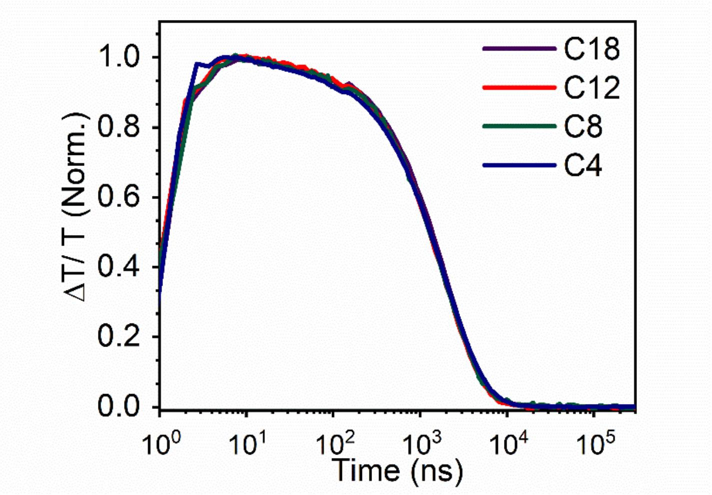

Figure S1 TA kinetics of PbS QDs with various alkyl ligands at 920 nm with an excitation at 1064 nm (in the absence of TES-ADT)

Table S1. Fitting results in TA decays of PbS QDs (Figure S1)

The number of carbon atom in the ligand	τ (ns)
18	2292 ± 14
12	2225 ± 42
8	2288 ± 48
4	2225 ± 24

TTA PUC emission from TES-ADT/ PbS QDs system

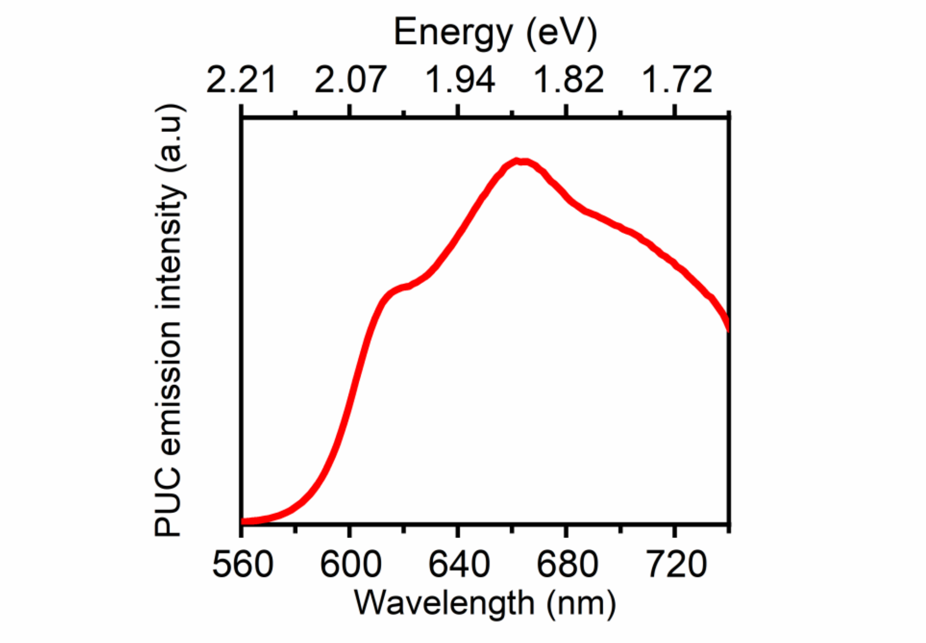

Figure S2. PUC emission from TES-ADT (100 mM)/ PbS QDs with the C12 ligand in toluene with an excitation at 1064 nm

Lifetimes of PbS QDs in the presence of Rubrene molecules

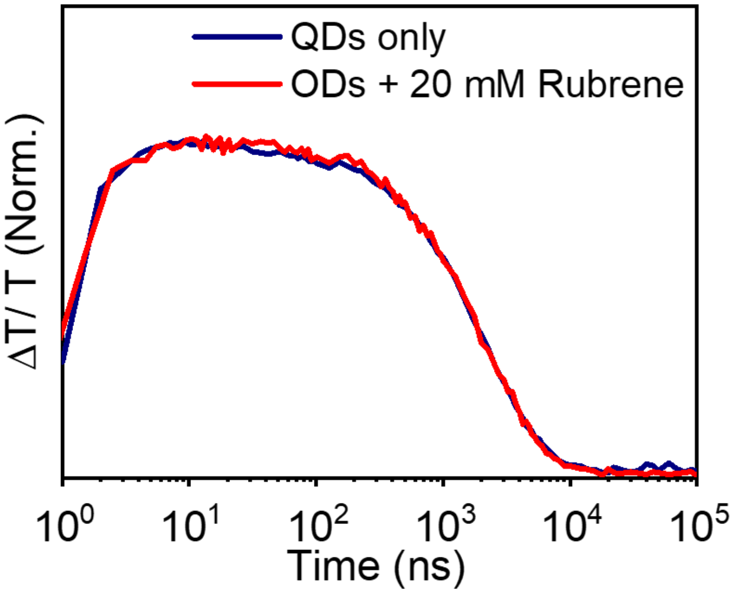

Figure S3. TA kinetics of PbS QDs with the C18 ligand in the presence (red) or absence (blue) of 20 mM Rubrene at 920 nm with an excitation at 1064 nm

Table S2. Fitting results in TA decays of PbS QDs in the absence or presence of Rubrene (Figure S3)

in the presence/absence of 20 mM Rubrene	τ (ns)
Absence	2292 ± 14
Presence	2303 ± 23

Quenching of PbS QDs in the presence of TES-ADT

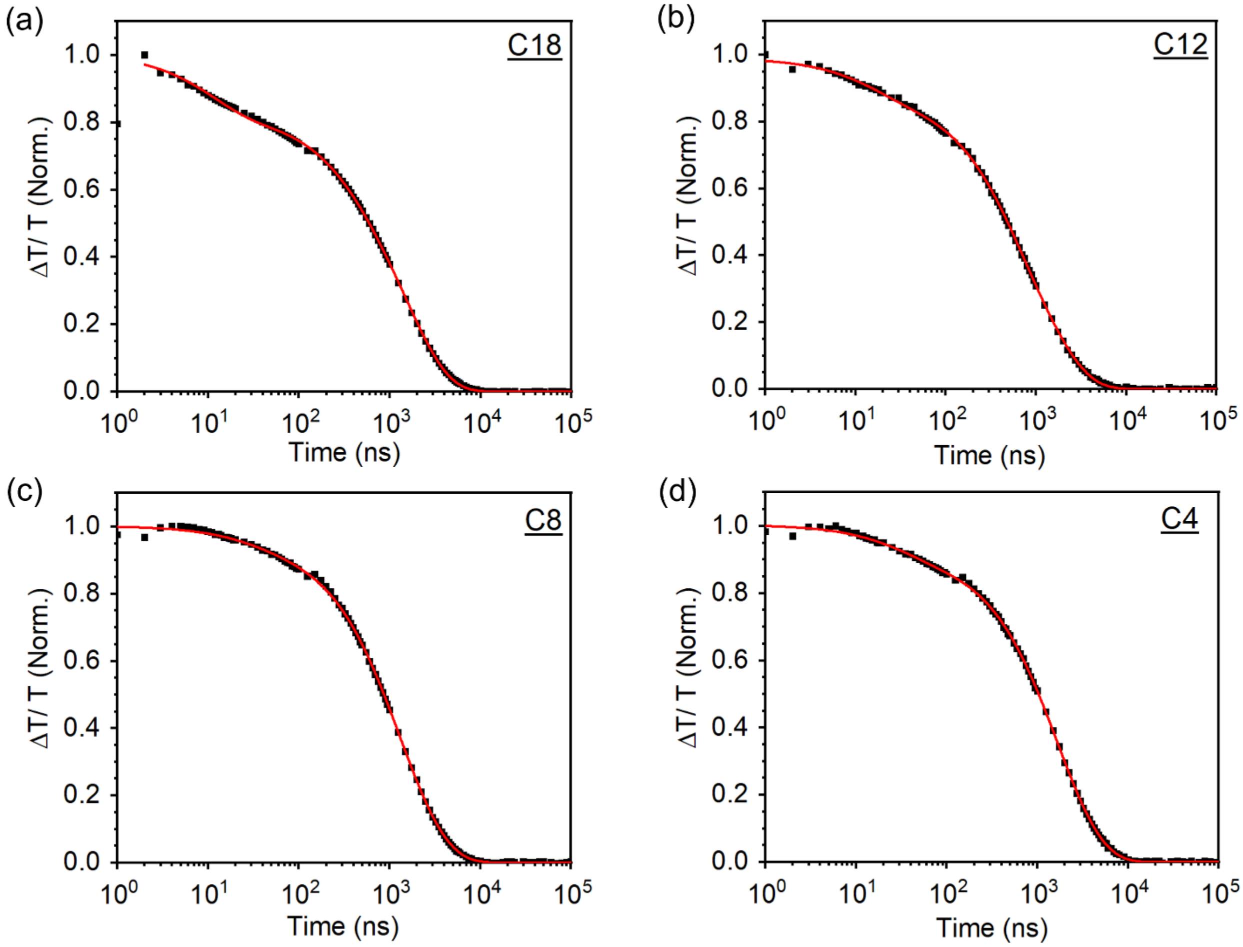

Figure S4. Fitting curves of TA decays at in TES-ADT/PbS QDs system with (a) C18 ligand, (b) C12 ligand, (c) C8 ligand, (d) C4 ligand

Table S3. Fitting results in TA decays of TES-ADT/PbS systems at 980 nm (Figure 3c and S4)

Ligand	A_1 (%)	A_2 (%)	A_3 (%)	τ_1 (ns)	τ_2 (ns)	τ_3 (ns)	$\tau_{av.}$ (ns)
C18	17	15	68	10.80 ± 0.47	352.1 ± 36.2	1676 ± 30	1195
C12	12	35	53	15.1 ± 0.9	432 ± 27	1500 ± 48	962
C8	6	36	58	30.2 ± 5.2	806 ± 103	1971 ± 132	1440
C4	30	24	46	30.2 ± 2.6	1023 ± 162	2311 ± 164	1349
